# Surface Preparation and Deposited Gate Oxides for Gallium Nitride Based Metal Oxide Semiconductor Devices

**DOI:** 10.3390/ma5071297

**Published:** 2012-07-24

**Authors:** Rathnait D. Long, Paul C. McIntyre

**Affiliations:** Department of Materials Science and Engineering, Stanford University, Stanford, CA 94305, USA; E-Mail: pcm1@stanford.edu

**Keywords:** Keywords: GaN growth, oxides, high-κ, surfaces, treatments, interface, ALD

## Abstract

The literature on polar Gallium Nitride (GaN) surfaces, surface treatments and gate dielectrics relevant to metal oxide semiconductor devices is reviewed. The significance of the GaN growth technique and growth parameters on the properties of GaN epilayers, the ability to modify GaN surface properties using *in situ* and *ex situ* processes and progress on the understanding and performance of GaN metal oxide semiconductor (MOS) devices are presented and discussed. Although a reasonably consistent picture is emerging from focused studies on issues covered in each of these topics, future research can achieve a better understanding of the critical oxide-semiconductor interface by probing the connections between these topics. The challenges in analyzing defect concentrations and energies in GaN MOS gate stacks are discussed. Promising gate dielectric deposition techniques such as atomic layer deposition, which is already accepted by the semiconductor industry for silicon CMOS device fabrication, coupled with more advanced physical and electrical characterization methods will likely accelerate the pace of learning required to develop future GaN-based MOS technology.

## 1. Introduction

Gallium nitride (GaN) is a wide bandgap (3.4 eV) semiconducting material with a high breakdown voltage and, as such, is ideal for high frequency, high power and high temperature applications [1]. The use of GaN in commercial electronic devices outside the LED market has until recently been somewhat limited. However, GaN-based high electron mobility transistors (HEMTs) have been commercially available since 2006 and have been used in various wireless applications. The requirement, until relatively recently, that GaN epitaxial layers be grown on a sapphire or SiC substrate has been an economic barrier to more widespread adoption of GaN in many technological applications.

In 1993, Morkoc remarked that for GaN to become an important semiconductor material for large-scale manufacturing of electronic devices ‘GaN, despite fundamentally superior electronic properties and better Ohmic contact resistances, must overcome the lack of an ideal substrate material and a relatively advanced SiC infrastructure in order to compete in electronics applications’ [2]. Today such development is ongoing. By the early 2000’s growth of GaN onto silicon wafers was being routinely reported in the research literature, despite the large lattice mismatch of 17% (and thermal expansion coefficient mismatch of 56%) between the two materials. Today GaN-on-Si is commercially available from a number of sources [3,4,5]. Indeed the ability to produce bulk GaN single crystal wafers has also progressed, with a breakthrough in the resulting crystal quality recently reported [6]. The cost of using GaN wafers as substrates for GaN-based devices is still prohibitive for practical device technology. It is possible however, that this cost will reduce over time, driven largely by strong growth in demand in power electronic applications as well as major improvements in the process tools for production [7]. For high power applications in particular, GaN-on-Si or bulk GaN wafers are preferred to a GaN layer grown on a thermally insulating substrate for increased heat dissipation.

Power transistors are used as a switching element in power supply circuits, for example, in inverters. Inverters are able to control electric motors by converting the frequency of the AC power supply and are thus widely used for energy-efficient motor control. Therefore, great commercial potential motivates research and development that would enhance the performance of power transistors for higher energy efficiency systems. Currently, Si-based insulated gate bipolar transistors (IGBTs) are used as power transistors; unfortunately, their performance deteriorates significantly at high temperatures with a reduced operation current above 200 °C. This is largely attributable to the modest bandgap of Si (1.12 eV). This provides the motivation to develop power transistors using a new semiconductor (e.g. GaN with a bandgap of 3.4 eV), in order to realize a large output power and small on-resistance at high operating temperature [8].

**Figure 1:**
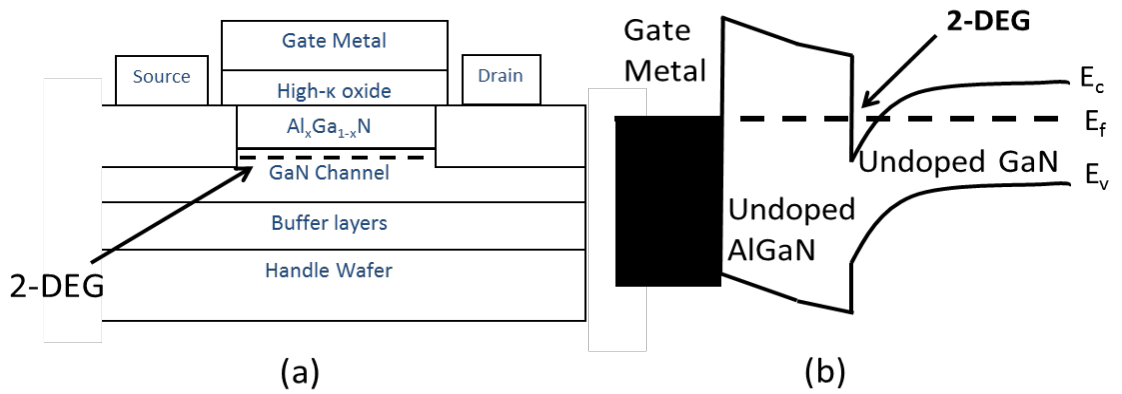
(**a**) Example of a commercial AlGaN/GaN high electron mobility transistors (HEMTs) structure; (**b**) Band Structure.

There are two specific power device structures for which GaN-based devices are becoming increasingly relevant – the metal-insulator (oxide)-semiconductor field effect transistor (MISFET or MOSFET), and the aforementioned HEMT structure (shown in Figure 1a). The most prevalent HEMT structure is the AlGaN/GaN HEMT. The term “high electron mobility” is applied to the device because the structure takes advantage of superior transport properties of a two-dimensional electron gas (2DEG) in a potential well of a wide bandgap undoped semiconductor (AlGaN) material on a narrower band material (GaN). A sharp dip in the conduction band edge occurs at the AlGaN/GaN interface (shown in Figure 1b). This results in high carrier concentration in a narrow region (quantum well) parallel to the surface under the AlGaN layer. The distribution of electrons in the quantum well is essentially two-dimensional due to its very small thickness in comparison to the width and length of the channel.

**Figure 2:**
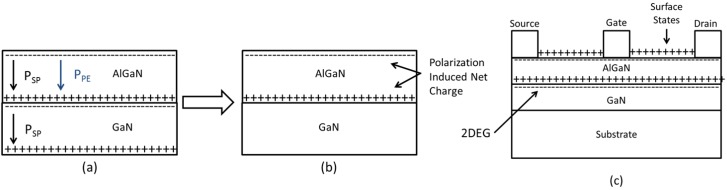
(**a**) Individual polarization charge contributions; (**b**) Polarization induced net charge; (**c**) the AlGaN/GaN HEMT with polarization induced net charge and surface charge contributions; from reference [10].

Polarization effects in AlGaN/GaN HEMTs include spontaneous and piezoelectric polarization (see Figure 2). The spontaneous polarization refers to the built-in polarization field present in an unstrained crystal. This occurs in both AlGaN and GaN independently because these Wurtzite-structure semiconductors are polar crystals (they lack inversion symmetry). Both materials have a [0001] polar axis, but their spontaneous polarizations are not identical because of the different compositions. The piezoelectric polarization results from elastic distortion of the crystal lattice. Due to the difference in lattice constant between AlGaN and GaN (2.4% difference between AlN and GaN at room temperature [9]), the AlGaN layer, which is grown on a relaxed GaN buffer layer, is biaxially strained (tensile strain). This strain contributes to a sheet charge on the two faces of AlGaN layer. The built-in static electric field in the AlGaN layer induced by the various polarization effects greatly alters the band diagram and the electron distribution of the AlGaN/GaN heterostructure [10] as illustrated in Figure 1b. The occupancy of surface states on the AlGaN surface influences the electrostatic boundary conditions on the 2DEG, controlling the density of carriers in the channel of the transistors.

Ibbetson *et al*. found that AlGaN surface states act as a source of electrons in the 2DEG [11]. For a non-ideal surface with available donor-like defect states, the energy of these states will increase with increasing AlGaN thickness. At a certain thickness, a state’s energy aligns with the Fermi energy level and electrons are then able to transfer from occupied surface states to empty conduction band states at the interface, creating a 2DEG and leaving behind positive surface sheet charge. For an ideal surface with no surface states, the only available occupied states are in the valence band. Here, the 2DEG exists as long as the AlGaN layer is thick enough to allow the valence band to align with the Fermi level at the surface. This means that in all cases, a positive sheet charge at the surface must exist in order for the 2DEG to be present at the AlGaN/GaN interface.

The surface states act as electron traps located in the access regions between the metal contacts (see Figure 2c). Successful surface passivation prevents the surface states from being neutralized by trapped electrons and, therefore, maintains the positive surface charge. If the passivation is imperfect, then electrons, leaking from the gate metal under the influence of a large electric field present during high power operation, can get trapped in the gate stack. The reduction in the surface charge due to the trapped electrons will produce a corresponding reduction in the 2DEG charge, and therefore reduce the channel current [10,12]. SiN_x_ is currently a common passivation for AlGaN/GaN HEMTs; it was initially reported by Kim *et al.* [13]. GaN cap layers (among others) have also been reported as passivating layers for AlGaN surfaces [14,15].

There are some limitations associated with these structures. Firstly, AlGaN/GaN HEMTs intrinsically show a negative threshold voltage, resulting in normally-on operation. Thus they need a drive circuit to control the gate bias, which can result in increased circuit complexity and higher costs. Especially in view of power device applications, a normally-off type transistor in which no current flows at 0V gate bias is strongly desired for fail-safe operation as well as reduced power consumption [16]. Secondly, high gate leakage current limits the performance of the device [17,18,19]. To date GaN HEMTs have primarily been used in radio frequency (RF) amplifiers for base stations and military applications. In such RF devices there is also a need to reduce gate leakage current, High-κ insulators/oxides on the AlGaN/GaN HEMT structures have been used to address this problem—this aspect will be discussed later in this review.

**Figure 3:**
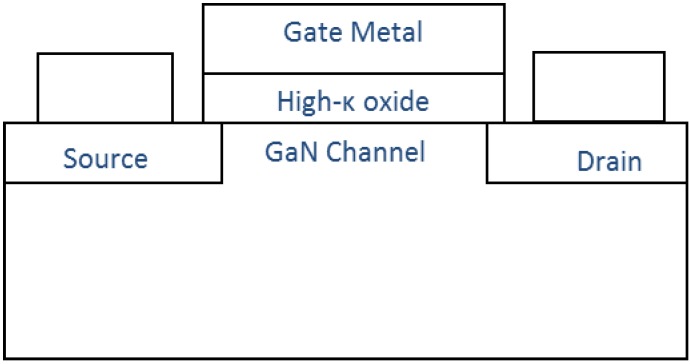
Structure of GaN based metal oxide semiconductor field effect transistor.

The second structure of interest for power applications is the metal-insulator (oxide)-semiconductor field effect transistor (MISFET or MOSFET), the structure of which is shown in Figure 3. While the MISFET does not benefit from enhanced electron mobility due to quantum confinement in a 2DEG at low gate bias, it does provide the capability of fabricating normally off devices with low gate-leakage current [20]. If GaN MISFETs can be fabricated with high field-effect mobility and high breakdown voltages, GaN MISFET technology might compete with SiC MOSFET technology [21]. Recently, a HfO_2_/GaN MOSFET which has the potential to rival state of the art GaN based HEMTs has been reported [22].

The increased understanding of oxide/GaN interface properties required for both HEMT and MOSFET devices coupled with the realization of GaN-on-Si technology motivates detailed investigations into all aspects of the GaN-insulator (oxide) based devices. From a technology adoption point of view, the International Technology Roadmap for Semiconductors (ITRS) points out the need for “reduction of leakage current and understanding of failure mechanisms, particularly for GaN materials which are piezoelectric in nature” [23].

This review will, therefore, summarize results reported in the literature on the preparation of GaN surfaces for subsequent deposition of gate insulators, the resulting interfaces between the GaN and the insulator (oxide) and the subsequent electrical characteristics of the GaN based metal oxide semiconductor (MOS) devices. Section 2 focuses on various growth techniques for GaN and investigations of the resulting material properties. Section 3 examines the ability to modify the GaN surface with *in situ* and *ex situ* treatments. Section 4 focuses on reported electrical properties of the GaN based MOS devices.

## 2. GaN Surfaces

GaN can exist in either a cubic crystal structure (zinc blende phase) or a hexagonal (Wurtzite) crystal structure [24]. The polar hexagonal crystal structure is of most technological interest [25]. Therefore, polar GaN is the relevant surface for this review. The polar surface with Ga face termination is represented by the Miller indices (0001) and the polar surface with N face termination is represented by the Miller indices (000). Because GaN has relatively large ionicity, the ionic character of the Ga-N bond and associated electrostatic effects can be expected to play an important role in its structural and electronic properties, which are not as well understood as those of low-ionicity semiconductors such as GaAs [26]. In Wurtzite GaN-based heterostructures grown in the typical (0001) direction, charges trapped in surface and interface states, spontaneous and piezoelectric polarization (the latter in the presence of strain) all contribute to a total charge density at the hetero-interfaces of up to several 10^13^ cm^−2^ [27].

### 2.1. Growth of GaN

The growth of GaN thin films can occur via a number of techniques: molecular beam epitaxy (MBE), metal organic chemical vapor deposition (MOCVD, also referred to as metal organic vapor phase epitaxy—MOVPE) and hydride vapor phase epitaxy (HVPE) are commonly-reported methods. Developments of each of these growth methods is still very much ongoing [28,29,30,31,32]. Depending on the growth mechanism and deposition conditions, significantly different GaN surface properties can be observed, both structurally and electronically. Structurally, the growth mechanism can affect the surface roughness and top layer termination. The top layer termination and electronic structure in turn affect the Fermi level (the highest energy level that an electron can reach or occupy in a material at absolute zero temperature) at the semiconductor surface. This is a critical factor for the electrical performance of a fabricated device. If a large number of similar defects are present on the GaN surface, the Fermi level becomes pinned (unable to move under an applied bias due to continuous charging of a high density of defects with energies in the bandgap) [33]. For a metal/semiconductor system, this may be desired if pinning occurs near the majority carrier band of the semiconductor; however, for MOS based devices or HEMT devices, the ability to modulate the charge at the surface of the semiconductor (and thus move the Fermi level) is of critical importance.

MBE is the one of the most widely reported methods of GaN epitaxial layer growth. Molecular beam epitaxy takes place in high vacuum or ultra-high vacuum, which allows *in situ* monitoring of the growth process. The most important aspect of MBE is the slow deposition rate (typically less than 1000 nm per hour), which promotes growth of epitaxial films on appropriate substrates. In solid-source MBE, elements such as gallium and arsenic are heated in separate effusion cells until they begin to slowly sublime. The gaseous elements then condense on the wafer, where they may react with each other. The term “beam” means that evaporated atoms are confined to a small solid angle as they travel from the effusion cell to the substrate, and they do not interact with each other or vacuum chamber gases until they reach the wafer. This is, in part, a consequence of the long mean free paths of atoms in high vacuum or UHV environment. In the GaN MBE process, the source of the nitrogen can either be ammonia or N_2_, with atomic N generated using an RF plasma [34]. The growth of MBE GaN can occur on various substrates, for example sapphire or SiC.

**Figure 4:**
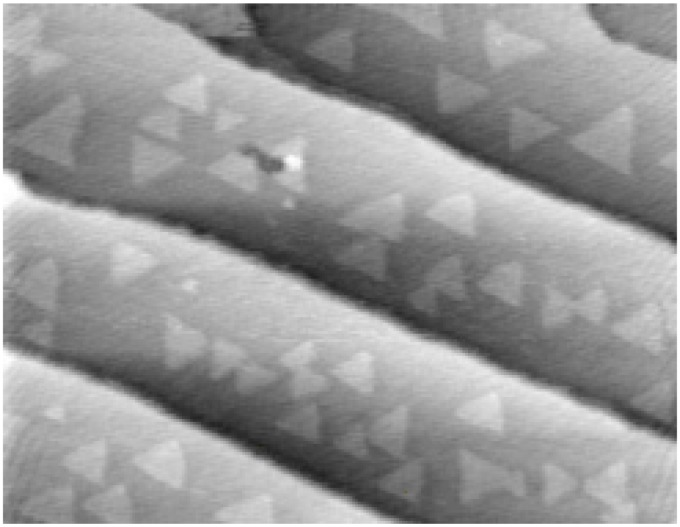
Scanning tunnelling microscopy (STM) image of triangular island formation during MBE growth in Ga rich conditions of (0001) GaN on SiC substrate [39]. Reprinted with permission from reference 39. Copyright (2006) by the American Physical Society.

There is a consensus, supported by both theoretical and experimental results [35,36,37], that it is better to perform the MBE growth of GaN under Ga-rich conditions to achieve a stable surface with minimal surface roughness. From a structural point of view, nucleation of GaN during MBE growth shows a novel behavior under excess-Ga deposition conditions. STM images show ‘ghost’ islands whose overall island density is found to depend critically on the surface Ga coverage [38]. Xie *et al.* [39] suggest, based on the two-dimensional island shape and surface step structures (as shown in Figure 4), that the growth is atom attachment limited under the excess-Ga condition but diffusion limited in the excess-N regime. Calculations reported by Takeuchi *et al.* [40] suggest that there is a clear reduction of N and Ga surface diffusion barriers at high Ga coverage on both Ga face and N face, consistent with the experimentally observed improved morphology of the material grown under the Ga rich conditions. However, there may be some drawbacks when growing in a Ga-rich MBE process—a scanning current–voltage microscopy (SIVM) study revealed that samples grown by MBE under Ga-rich conditions show three orders of magnitude higher reverse bias leakage compared with those grown under Ga-lean conditions [41]. The authors concluded that the high reverse bias leakage was predominantly observed at dislocations with a screw component. It is important to note here, that Ga-rich growth refers to the amount of Ga available during the growth process, as opposed to formation of a Ga-rich surface, which would be more metallic in nature (and may have a pinned Fermi level). Understanding dislocation formation in GaN is very necessary and still in progress. Moram *et al.* have recently investigated how different types of threading dislocations can result in different GaN microstructure, and discussed how threading dislocation mobility as well as the generation process, should be considered [42].

Adelmann *et al.* [43] used reflection high energy electron diffraction (RHEED) and first-principle growth models to quantitatively determine the Ga coverage on the GaN (0001) surface as a function of Ga flux and substrate temperature during MBE. They also derived a model, which describes the adsorption of Ga on GaN surfaces as well as the accumulation of Ga on the surface during Ga-rich GaN growth. Such studies are important for understanding the mechanisms dominating the growth processes and subsequently how they can be manipulated to achieve certain surface characteristics. The authors of reference [36] show that experimental results on Ga adsorption and desorption on Ga-polar GaN can be rationalized using the Wolkenstein theory for adsorption on semiconductors [44] in which the surface Fermi level and/or the surface charge plays a dominant role in adsorbate chemisorption. They also show that because of the fixed polarization charge existing at the GaN (0001) surface, Ga adsorption and desorption processes involve both neutral and charged Ga states (due to charge transfer between the polar Ga face GaN surface and the Ga adsorbate). This work is an example of how the study of GaN differs significantly to other 3-V materials such as GaAs because of the large polarization effects in the former material.

Recently, higher temperature (~780 °C) MBE coupled with a lower Ga/N flux ratio was shown to have advantages compared to the more common lower temperature (650–740 °C) Ga-rich MBE. The major benefit of this process was a much wider growth window for smooth GaN without excess metallic Ga. Higher-quality epilayer growth near Ga/N = 1 resulted from lower thermal decomposition rates of the GaN and was corroborated by slightly lower (inferred) Ga vacancy concentrations, lower unintentional oxygen incorporation, improved electron mobilities and reduced densities of leakage current paths compared to low-temperature grown GaN films [28].

MOCVD of GaN is also regularly reported in the literature. MOCVD is a chemical vapor deposition method of epitaxial growth of materials from the surface reaction of organic compounds or metalorganics and hydrides containing the elements of which the desired thin film is composed. Formation of the epitaxial layer occurs by pyrolysis of the constituent chemicals at the substrate surface. In contrast to MBE, the growth of crystals accompanies chemical reactions, and is a purely physical process of condensation. Chemical vapor deposition takes place not in a vacuum, but from gas phase precursors at moderate pressures (2 to 100 kPa). MOCVD-grown GaN is routinely used in device fabrication because the growth rates are typically higher than for MBE, and because the films are reported to yield superior optoelectronic devices. In some cases, the MBE GaN surface structure can be similar to that of the MOCVD GaN, for example, as reported by Manske *et al.* [45]. These authors used low energy electron diffraction (LEED) to observe nanosized clusters on both surfaces irrespective of polarity and the starting MOCVD grown GaN which was from three different sources. However, significant differences between MOCVD and MBE GaN layers are also reported. MOCVD-grown GaN with a Si dopant concentration above 1 × 10^19^ cm^−3^ has been reported to exhibit crack formation [46] and residual tensile stress [47]. On the other hand, Si doping of GaN layers grown by MBE has been shown to lead to smooth surfaces [46]. Although some work specifically examining the MOCVD GaN surface has been carried out [48,49,50], more research is required in order to link the GaN surface structure to the properties of fabricated electronic devices.

In the HVPE process, Group 3 nitrides such as GaN are formed by reacting hot gaseous metal chlorides (GaCl) with ammonia gas (NH_3_). The metal chlorides are generated by passing hot HCl gas over heated Group 3 metals. All reactions are done in a temperature controlled quartz furnace. Unlike MOCVD, the HVPE process does not involve metalorganic precursors, thus providing a ‘carbon-free’ environment for epitaxial growth. In addition, the use of gaseous hydrogen chloride can provide an intrinsic impurity cleaning effect by partially etching the film surface as it is deposited. This results in epitaxial layers with low background impurity concentrations and more efficient doping control. A key feature of HVPE is its high growth rate (at up to 100 µm per hour), almost two orders of magnitude faster than typical MOCVD and MBE processes. However, this faster growth rate can come at a price of reduced reproducibility [51,52]. While the quality of the HVPE GaN is improving, there are presently a much smaller number of examinations of HVPE grown surfaces and the electrical properties of resulting devices compared to MBE and MOCVD GaN surfaces [53,54].

### 2.2. Surface Reconstruction

Gallium nitride surface reconstruction may contribute directly to the interface defect density of MOS devices. Several scanning tunnelling microscopy (STM) studies have been carried out for different GaN surfaces [55,56,57]. Smith *et al.* [58] identify 2 × 2, 5 × 5 and 6 × 4 reconstructions on GaN (0001) surfaces. With increasing Ga coverage, the 5 × 5 and 6 × 4 structures are formed. For metal oxide deposition techniques in which nucleation on the GaN surface is critical to the subsequently formed oxide/semiconductor interface, understanding the starting surface structure is important. Techniques such as STM have the potential for identifying the structure of surface defects and, by implication, interface defects (e.g. dangling bonds) that degrade electrical performance. They may also provide insights into approaches for effective interface state passivation.

### 2.3. Bulk Defects

Effects of the growth process on the electronic structure and performance of semiconductor devices fabricated in the GaN epilayers should not be underestimated [59]. The key quantities that characterize a defect in a semiconductor are its concentration and the position of its transition levels (or ionization energies) with respect to the band edges of the material [60]. One aim, therefore, of tuning the GaN bulk structures and compositions through variation of epilayer growth conditions is ultimately to reduce the number of electrically active defects in the bulk of the GaN. In the following paragraphs, results reported on the identification of bulk defects in GaN, using both theoretical and experimental methods will be presented. The focus is kept on work that discusses direct identification of defect energies in the GaN bandgap, or the energy in the GaN bandgap at which the Fermi Level is pinned (unable to be modulated) due to high defect densities.

The position of the defect transition levels with respect to the GaN band edges determines their effects on the electrical and optical properties of this semiconductor. Defect formation energies and transition levels can be predicted from first principles [60]. First principles calculations based on density functional theory (DFT) offer the most accurate theoretical description of structural and electronic properties of the GaN surface. Unfortunately, many of these studies suffer from the underestimation of the band gap and excited state energies inherent to DFT in the local density approximation, which complicates the study of surface electronic structure [61]. Pollmann *et al.* [57] and Segev and Van de Walle [58] demonstrate corrected methods including relaxation which can be adopted in order to obtain the experimentally observed 3.4 eV [49] band gap of GaN, allowing more accurate representations of the real system.

**Figure 5:**
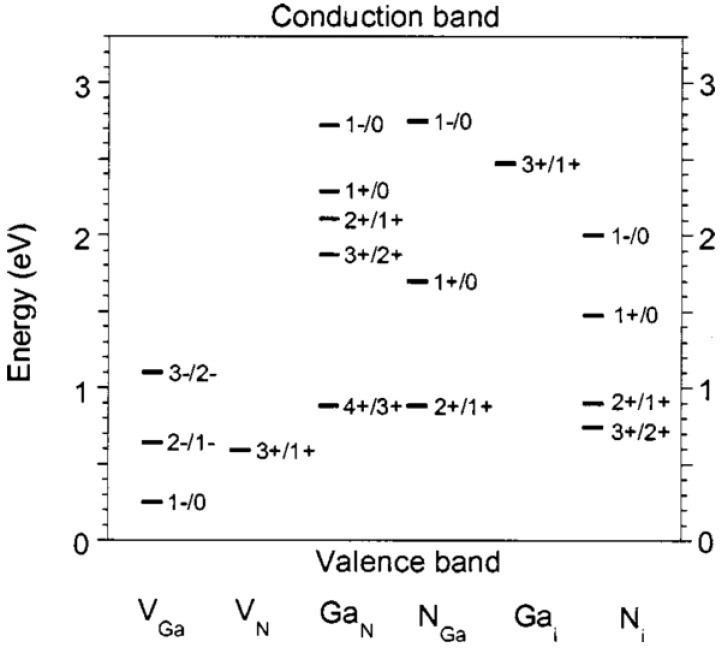
Transition levels for native point defects in GaN. Defects in semiconductors and insulators can occur in different charge states. For each position of the Fermi level, one particular charge state has the lowest energy for a given defect. The Fermi-level positions at which the lowest-energy charge state changes are called transition levels or ionization energies. For example, from this figure Ga vacancies are predicted to be in the −3 charge state for Fermi levels greater than 1.1 eV above the valence band edge [63]. Reprinted with permission from reference 63. Copyright (2005) by the American Institute of Physics.

Van de Walle and Segev have calculated the energy levels of major bulk point defects in Wurtzite phase GaN [62], and the results are summarised in Figure 5 [63]. When examining this figure, it is important to realize that (a) not all of these defects will be present at the surface of a GaN epilayers; and (b) the densities of some of these defects may be small enough to not have an impact electrically. Experimental data on the energy levels of bulk point defects, and on their formation and removal from GaN have also been reported [64,65,66], mainly using Deep Level Transient Spectroscopy (DLTS) and Deep Level Optical Spectroscopy (DLOS). These techniques allow defects present at energies throughout the entire bandgap of the GaN to be examined—DLTS probes within 1 eV of the conduction band minimum, while DLOS probes from ~ 1 eV below the conduction band minimum to the valence band maximum. Using this technique the authors report that there are bulk carbon-related defects as well as bulk Ga vacancies with energies near the valence band maximum. The defect associated with N vacancies is near the conduction band edge. Petravic *et al.* [64] suggest that argon bombardment may preferentially remove nitrogen from GaN thus creating an excess of nitrogen vacancies. The argon bombardment produces a metallic Ga layer at the surface, which results in increased band bending and pinning of the surface Fermi level close to the conduction band minimum. Even without argon bombardment these native donor defects can be responsible for the n-type conductivity of as-grown undoped GaN. The authors of reference [64] demonstrated the presence of nitrogen interstitial electronic states within the GaN band gap, in agreement with theoretical predictions. The reduction in band bending determined from photoemission measurements was consistent with the expected acceptor-like character of these defects.

### 2.4. Surface Defects

The surface of GaN may contain defects related to some of the bulk defects just described. Some of these may be surface dangling bonds. In addition defects produced by native oxide formation and/or by reaction with adsorbates will be present. Tuning the growth conditions can also influence the surface of the GaN layer as well as its bulk properties. In reference [67], Van de Walle carried out calculations focusing on surface states and showed that at typical (close to stoichiometric) Ga/N surface ratios, the Fermi level is pinned at 0.5–0.7 eV below the conduction-band minimum (CBM) for the (0001) surface and at 1.2 eV above the valence-band maximum (VBM) for the (000) surface plane. For highly Ga-rich conditions, the Fermi level is calculated to be 1.8 eV above the VBM for the (0001) and 1.6 eV for the (000) plane. The results are in agreement with experiment, for example see reference [68] for the Ga rich (000) case, and suggest that the microscopic origin of the Fermi-level pinning on GaN surfaces occurs via formation of either Ga dangling bonds at moderate Ga/N ratios or Ga-Ga bonds on Ga-rich surfaces. Reference [27] gives similar pinned surface Fermi levels determined experimentally using xray photoelectron spectroscopy for these substrate planes.

Table 1 summarizes the defect energy levels presented in this section. In Section 3, the presented defect levels will be discussed further with regard to experimental interface defect density measurements obtained from GaN MOS devices.

**Table 1:** Summary of relatively recent literature on the defect energy levels presented in this review.

Reference	GaN Growth technique	Defect level	Defect type	Defect origin	Method of evaluation	Comment
[65]	Ammonia MBE n type GaN	E_c_ − 3.28	Bulk	Carbon related acceptor impurity	DLTS/DLOS	Also observed for plasma assisted MBE and MOCVD GaN
		E_c_ − 2.62	Bulk	Gallium Vacancy	DLTS/DLOS	–
		E_c_ − 1.28	Bulk	Carbon related interstitial impurity	DLTS/DLOS	Also observed for plasma assisted MBE and MOCVD GaN
		E_c_−0.72	Bulk	Unknown	DLTS	Observed in MBE & MOCVD GaN; low concentrations
		E_c_−0.62	Bulk	Unknown	DLTS/DLOS	Seen in alternative growth methods; not carbon related
		E_c_ − 0.4	Bulk	Unknown	DLTS	Observed in MBE & MOCVD GaN; low concentrations
		E_c_ − 0.25	Bulk	Nitrogen vacancy related	DLTS/DLOS	Seen in alternative growth methods
[66]	MOCVD n type GaN	E_c_−E_t_ = 3.28	Bulk	Carbon related acceptor impurity	DLTS/DLOS	–
		E_c_−3.22	Bulk	Acceptor	DLTS/DLOS	Not seen in MBE grown GaN
		E_c_ − E_t_ = 1.35	Bulk	Carbon related interstitial impurity	DLTS/DLOS	–
[64]	MOCVD p type GaN	–	Surface	Acceptor N Interstitials	NEXAFS	–
		Pin Fermi Level close to CBM	Surface	Donor N vacancies	NEXAFS	–
[67]	N/A	Fermi level pinned 0.5–0.7 below CBM on polar Ga face	Surface	Ga dangling bonds	Modified DFT	N Type SubstrateModerate Ga/N ratios (close to 1)
		Fermi level pinned 1.2 above VBM on polar N face	Surface	Ga dangling bonds	Modified DFT	P Type SubstrateModerate Ga/N ratios (close to 1)
		Fermi level pinned 1.8 above VBM on polar Ga face	Surface	Ga-Ga bonds	Modified DFT	N Type SubstrateGa rich conditions
		Fermi level pinned 1.6 above VBM on polar N face	Surface	Ga-Ga bonds	Modified DFT	P type SubstrateGa rich conditions
[27]	MBE n type GaN	E_f_ − E_VBM_ = 2.89eV	Surface	–	XPS	Moderate Ga/N ratios (close to 1)
		E_f_ − E_VBM_ = 1.65eV	Surface	–	XPS	Ga rich conditions

## 3. GaN Surface Treatment

Most reported investigations into the effect of surface treatments on GaN have been carried out during the fabrication of metal-GaN Schottky contacts, where an important aim is to remove surface contaminants such as oxygen and carbon. Many of the results summarized here are from published reports of such studies. The surface chemistry, the electronic structure of the GaN surface and its atomic structure are important and interrelated factors when reviewing a cleaning process. For metal-polar Ga face GaN interfaces, the performance of the contact depends sensitively on the starting GaN surface. This surface has usually being exposed to atmosphere, thus a native oxide forms which can lead to contact problems in these structures [69,70]. Edwards *et al.* [71] report the existence of an overlayer on the GaN surface of 2–5 nm thickness, half of which consists of organic and inorganic contamination. The residual material in this layer is assumed to be native oxide. Oxygen at the GaN surface generally bonds to the Ga, consistent with large electronegativity difference for these two elements. However, some N-O bonding cannot be ruled out [72,73]. In examining the effect of a cleaning procedure, any unintentional deposition of species from the cleaning environment must be considered, as well as the ability of the cleaning chemistry to remove carbon and oxygen from the GaN surface. Table 2 gives the specific details on some of the surface treatments reviewed in this section. This is not meant to be a complete review of all cleans reported in the literature—but more of a summary of the types of cleans that have already been employed and examples of the subsequently observed GaN surface properties. Unless otherwise specified, the cleans were carried out at room temperature.

Diale *et al.* [74] report that surfaces cleaned in aqueous (NH_4_)_2_S give the lowest values of both C and O, RMS roughness and a Ga/N ratio closest to 1, in comparison to cleans in KOH and HCl. Nearly complete removal of C and O was achieved by subsequently heating the samples in an Auger Electron Spectrometry (AES) chamber in vacuum. Using (NH_4_)_2_S prevented re-oxidation of the surface (also described in reference [68]), and further removed Cl from the surface of the GaN, while KOH effectively removed C from the surface. The authors of [69] also report that (NH_4_)_2_S solution exposure immediately prior to e-beam deposition of a Pt/Au rectifying contact metal reduces the barrier height (on both p-type and n-type GaN), suggesting that minimizing oxide formation at the metal/semiconductor interface improves Ohmic contact resistance on GaN. Moreover, the surface recombination velocity of n-type heteroepitaxial GaN (0001) grown by HVPE on a sapphire substrate is found to decrease dramatically when the surface is chemically treated with aqueous and alcohol solutions of inorganic sulfides, such as ammonium or sodium sulfide ((NH_4_)_2_S_x_ and Na_2_S) [75]. The authors suggest that this is indeed due to reduced band bending associated with a reduced density of surface states on the treated surface.

The authors of [75] note that in general, acidic solutions increase the band-edge photoluminescence (PL) intensity (reduce the surface recombination velocity), whereas bases and H_2_O_2_ decrease the PL intensity. Acidic treatments such as HF can reduce the surface O concentration [76]. Lee *et al.* [77] report that UV/O_3_ and wet chemical treatments based on HF and HCl are very effective in removing surface C and reducing the native oxide thickness on GaN but it further suggests that high vacuum *in situ* cleaning methods are needed to obtain an oxygen free air exposed GaN surface. This highlights a potential problem, however, as *in situ* methods cannot be universally applied to commercial sample surfaces [78]. The authors of [79] studied the effects of GaN surface treatments on surface contaminants using STM. Images were obtained after cleaning the surfaces in acetone and isopropanol followed by aqueous HCl (1:1 HCl:distilled H_2_O), HF (2:1 HF:distilled H_2_O) or 2M NaOH exposure. Treatment in HF was the most effective method for removing surface oxygen (the authors of [78] do not specify the oxygen as being part of a native oxide—it may be present primarily in an adsorbate layer) and minimizing additional deposition of carbon or other surface contaminants. HCl treatment was equally effective at surface oxygen removal, but carbon and chlorine were also deposited on GaN during the cleaning procedure. Cleaning in 2M NaOH was more effective for removing surface carbon compared to HCl or HF; however, residual sodium remained on the surface and surface oxygen was still detectable.

**Figure 6:**
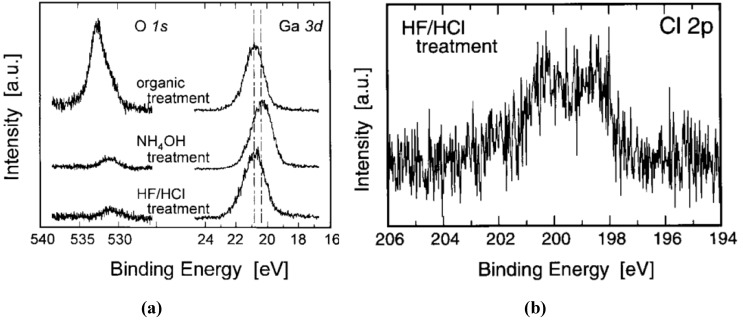
X-ray photoelectron spectroscopy (XPS) spectra of GaN surface following various aqueous cleaning treatments. In some cases the treatment may (**a**) achieve contaminant removal; but (**b**) create residues on the surface following the treatment [82]. Reprinted with permission from reference [82]. Copyright (1999) by the Elsevier.

Tracy *et al.* [80] report that a contamination layer of hydrocarbons and oxygen, the latter contained in a hydroxide, remained on aqueous HCl-treated surfaces. These authors demonstrated that *in situ* exposure of the surface GaN thin films to flowing ammonia at 860 °C and 10^−4^ Torr removes hydrocarbon and oxygen/hydroxide species below the detectable limits of X-ray and ultraviolet photoelectron spectroscopies and decreases the Ga/N ratio from 1.3 to 1.0. In contrast Grabow *et al.* [81] report that an NH_3_ treatment is efficient for the removal of carbon contaminants, but a complete removal of oxygen contaminants could not be achieved. Koyama *et al.* [82] report that the native oxide can be removed by an NH_4_OH(aq) treatment with no deposits left on the treated surface resulting in oxide-free and well-ordered surfaces, superior to those achieved with aqueous HF/HCl treatment, which leaves residual Cl on the surface (see Figure 6). These authors do not report the effect of wet cleans on carbon contamination. Table 2 presents a summary of the various GaN surface treatments presented in this section.

**Table 2:** More details on some of the cleaning procedures reviewed in this section.

Reference	GaN	Clean	Comment
[69]	Plasma Assisted MBE GaN (0001) on Sapphire	60 s in HCl (25 °C) and 30 s in buffered HF. The second cleaning process included a 20 min boil in (NH_4_)_2_S	boiling n- and p-GaN in (NH_4_)_2_S resulted in decreased barrier height
[74]	MOCVD GaN (0001) on Sapphire	(1) Degrease: Boil in trichloroethylene for 3 min; Boil in isopropanol for 3 min Three rinses in DI for 20 s each; Blow dry with N_2_; (2) Aqua regia (AR): Degrease; Boil in HCl:HNO_3_ = 3:1 for 8–10 min rinses in DI for 20 s each; Blow dry in N_2_; (3) HCl: Degrease; Aqua regia HCl:H_2_O = 1:1 dip for 60 s Two rinses in DI for 20 s each; Blow dry with N_2_; (4) KOH: Degrease; Aqua regia; 1 mol KOH boil for 3 min Three rinses in DI for 60 s each Blow dry with N_2_; (5) (NH_4_)_2_S: Degrease; Aqua regia; (NH_4_)_2_S for 1 min Three rinses in DI for 60 s each. Blow dry with N_2_	AES has shown the contaminant as C and O and that using compounds with Cl and S, will leave Cl and S on the surface. Using (NH_4_)_2_S prevented re-oxidation of the surface, and further removes Cl from the surface of the GaN. KOH effectively removes the C on the surface.
[68]	Plasma Assisted MBE GaN (000). on sapphire	Samples were rinsed in a 1:10 solution of HCl, and then rinsed in DI water. Cleaned by repeated cycles of nitrogen ion sputtering and annealing to 850 °C. Sulfur was deposited from an UHV compatible electrochemical cell.	Exposure of the clean Ga adlayer (000) GaN surface to sulfur inhibits oxygen adsorption
[75]	HVPE GaN (0001) on Sapphire & MOCVD GaN(0001) on SiC	Strong acids, strong bases, strong oxidizers. After dipping in a given chemical, the sample is then rinsed in DI water and solvents and blown dry with N2. Sulfide solution consisting of 20%–50% (NH_4_)_2_S and 50%–80% H_2_O.	Various chemicals strongly affect the surface recombination velocity in GaN. Treatment with (NH_4_)_2_S or Na_2_S effectively reduces the SRV in GaN. A 1:1 (NH_4_)_2_Sand CH(OH)CH_3_ solution increases the PL intensity by a factor of four to six. The effect of the non-sulfide treatments on the SRV is important for GaN device processing. The HCl, BOE, and HF treatments produce the greatest improvements.
[76]	MOVPE GaN (0001) on Sapphire	(1) Chemical Etch only:Degreased in acetone, dipped in HF: DI H_2_O 1:10 for 1 min, rinse in DI; (2) In situ Annealing: Chemical Etch; Annealed at 600 °C for 10 min in UHV; (3) *In Situ* Ga Reflux: Chemical Etch: 2 cycles of Ga deposition, reduction and re-evaporation in UHV; 900 °C anneal	All treatments result in surfaces increasingly free of oxide contamination. HF etching and UHV annealing produce abrupt, well-defined interfaces. Conversely, GaN substrate cleaning in a Ga flux results in Au/GaN intermixing.
[77]	MBE GaN (0001) on Sapphire	For ex situ cleaning, solvents (acetone and methanol), acids (HF and HCl), and UV/O_3_ treatments were used. The samples were rinsed in DI water and blown dry with N_2_. After all wet chemical treatments UV/O_3_ exposure was performed with N_2_ after all wet chemical treatments. N_2_ and N_2_/H_2_ plasma treatments were conducted in an ultrahigh vacuum (UHV) Metal Organic MBE	For ex situ cleaning methods, the lowest O levels on GaN surfaces were achieved after HF and HCl based acid treatments, while UV/O_3_ cleaning was very effective for removing carbon. Further removal of the O could be achieved after in situ thermal cleaning with H_2_/N_2_ plasma.
[78]	HVPE, LPE stand alone GaN (0001)	Rinsed in Ultrapure water for 5 min; dipped in etchant for 50–1000 s, rinsed in ultrapure water for 5 min, blown by dry N_2_, Four kinds of etchant were used: 0.5 wt.% HF (pH = 3.3 and ORP = 250mV), 0.7 wt.% HCl (2.1, 250 mV), 0.6 wt.% HNO_3_ (1.5, 240 mV), and 0.7 wt.% NaOH (12.0, −70 mV). All wet cleaning procedures were done under fluorescent light. Some samples were postannealed in UHV (base pressure: 1 × 10^−8^ Pa) by three-step annealing (200 °C for 12 h, 400 °C for 1 h, and 550 °C for 10min)	The HF-treated and postannealed commercial and original GaN (0001) could induce the similar quality to that of *in situ* MBE-grown GaN (0001) 2 × 2-N. In the case of performing wet etching on a GaN system, selecting an etchant solution with a certain pH and ORP and controlling etching time are important to obtain an oxide-free and balanced-stoichiometry surface.
[79]	MOCVD GaN (0001) on Sapphire	Boiling acetone followed by boiling isopropanol for 10 min each prior to HF (2:1 HF : distilled H_2_O), hot HCl (1:1 HCl: distilled H_2_O at 70 °C) or 2M NaOH.	Treatment in HF was the most effective method for removing surface O and minimizing additional deposition of C or other surface contaminants. HCl treatment was equally effective at oxygen removal, but C and Cl were also deposited during the cleaning procedure. The cleaning in 2M NaOH was more effective for removing surface C compared to HCl or HF; however residual Na remained on the surface and surface O was not removed.
[80]	MOVPE GaN (0001) on SiC	The samples were immersed in TCE, acetone, and methanol for 1 min in each solvent; in 37% HCl for 10 min and in de-ionized water for 10 s. In situ chemical vapor cleaning: Ammonia introduced into the chamber at a pressure of 1 × 10^−4^ Torr and the sample heated to 865 °C for 15 min. The sample was cooled to 500^o^C and then the ammonia was turned off.	A contamination layer of hydrocarbons and oxygen, the latter contained in a hydroxide, remained on the HCl treated surfaces. In contrast, the ammonia-based CVC produced surfaces that were free of these contaminants within the limits of XPS and UPS detection.
[81]	HVPE GaN (0001) on Sapphire	Degrease in an ultrasonic bath for 10 min each in TCE, acetone, methanol and DI water, then treated in concentrated HCl for 20 min, rinse in DI water, blow dry N_2_. NH_3_ introduced to the chamber at a pressure of 1 × 10^−4^ Torr. Sample was heated to 900^o^C for 20 minutes. Cooled and NH_3_ flow stopped when temperature reached 500^o^C	High temperature NH_3_ annealing treatment of the GaN (0001) surface can be used to clean the surface from carbon contaminants down to XPS detection limits, whereas a significant amount of residual oxygen species remains.
[82]	MBE GaN (0001) on Sapphire	(1) Cleaning in organic solvents; (2) Dip in HF:HCl:H_2_O = 1:5:5 solution for 1 min after organic solvents; (3) dip in NH_4_OH solution for 15 min at 50 °C	From the viewpoints of oxide removal and maintenance of stoichiometry, it is concluded that etching in NH_4_OH solution at 50 °C gives the best result.

It is clear that a good body of work has been carried out investigating the effects of various surface treatments on GaN surface contaminants and stoichiometry. However, the following points should not be ignored. Firstly, there appears to be, as yet, no dedicated investigation or discussion of the mechanisms by which these treatments actually reduce the amount of oxygen, carbon or other contaminants from the surface of the GaN. Secondly, a systematic examination of the effects of the GaN surface termination on contaminant and native oxide removal has not been reported. Thirdly, a note of caution: for metal-GaN contacts the interface requirements may be very different from those for oxide-GaN interfaces needed in field effect devices. One example of this is the desirability of different residual surface species on GaN used to fabricate metal/GaN and metal oxide/GaN interfaces. If residual Cl exists on the GaN surface for example—for a GaN-metal interface it’s presence may enhance the adhesion of the metal to the semiconductor surface [74]. However, for an oxide-GaN interface such species may produce unwanted electrically active defects at the interface or in the oxide. An approach such as that taken in [78] using HF(aq) and a post annealing step to modify an air-exposed surface to produce specific reconstructions and high surface quality seems promising for fabrication of high performance GaN based devices. It is clear at this point that more investigation is required in order to identify a cleaning procedure which will lead to enhanced electrical performance for metal-insulator-GaN devices.

## 4. GaN MOS

This section will focus on research reported on metal oxide/GaN semiconductor devices. Unless otherwise specified, ‘GaN’ will be taken as Wurtzite-structure n-type (0001) GaN.

### 4.1. Capacitance-Voltage Measurements

**Figure 7:**
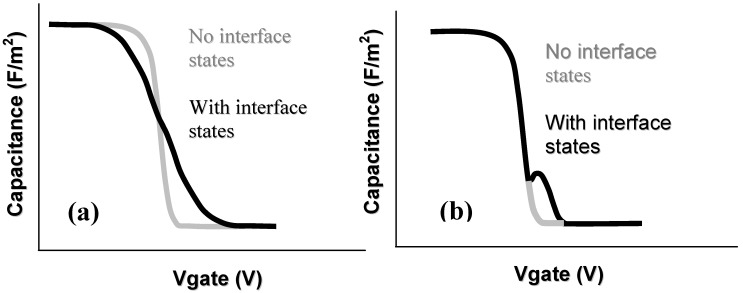
Ideal capacitance voltage profiles for a p-type semiconductor comparing with (**a**) stretch out of the profile due to a continuous energy distribution of interface defects; (**b**) profile modification due to electrically active defects with a narrow energy range at the dielectric-semiconductor interface.

Capacitance voltage (CV) measurements are the primary technique for evaluating the performance of any metal-oxide-semiconductor device. This measurement can be used in several different ways to extract an interface state density (D_it_)—the areal density of electrically active defects per unit energy within the semiconductor band gap at the oxide-semiconductor interface. Figure 7 shows the specific effects of the interface state density on the measured CV curve (for a p-type semiconductor); a stretch out of the CV characteristic along the gate voltage axis, and an addition of an interface trap capacitance to the overall measured capacitance of the MOS system, depending on the nature of the defects themselves. The stretch out of the CV response as shown in Figure 7a occurs due to the filling or emptying of interface defects as the dc bias on the gate is slowly varied, with the state filling or emptying depending on whether the defects are donor or acceptor in nature. This is as a result of a continuous distribution in energy of interface defects. For a peak energy distribution of interface defects an interface state capacitance will be added to the capacitive responses of the oxide and the semiconductor (e.g., in depletion) when the ac signal frequency and device temperature used in the capacitance measurement are such that the interface states can respond within the time constant of the ac signal. As a result, dispersion in the CV profile will occur as a function of the ac measurement frequency, displaying frequency dependent ‘peaks’ in the CV curve in the depletion/inversion regime, as shown in Figure 7b. The effect of the interface defects is to add a capacitive contribution C_it_ in parallel to the semiconductor differential capacitance as the interface defects contribute a rate of change of surface charge with respect to surface potential. Electrically active defects detected by capacitance voltage analysis would degrade MOSFET device performance e.g. increased subthreshold swing, threshold voltage instability, reduced carrier mobility *etc.* [83].

Capacitance voltage measurements are very well understood for the Si-SiO_2_ system [84]. They can provide a significant amount of information about defects in MOS gate stacks using capacitors (MOSCAPs) rather than transistors, avoiding possible processing-induced artifacts inherent in the more complex fabrication processes required for FETs, and significantly reducing device turn-around time. When applied to semiconductors such as GaN, however, there are a number of factors which should be considered.

**Figure 8:**
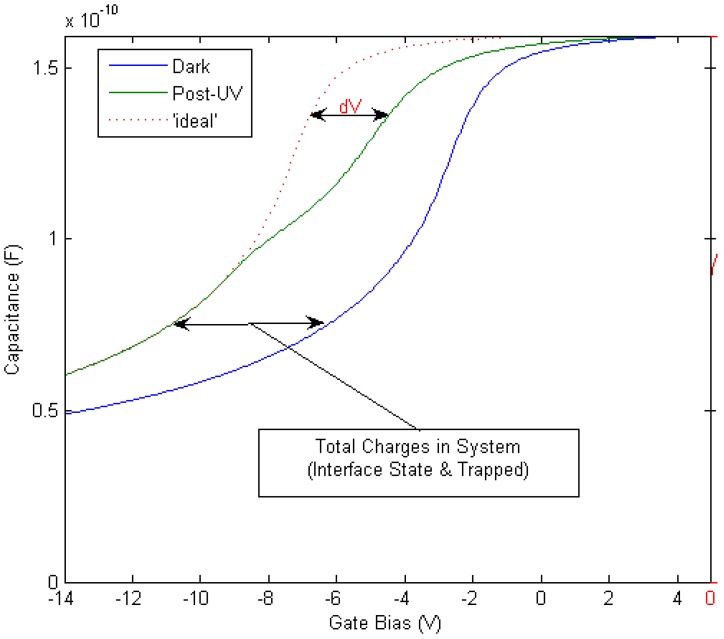
Modified Terman method for measurement of the interface state density using UV illumination [85]. Reprinted with permission from reference 85. Copyright (2009) by the American Institute of Physics.

**Figure 9:**
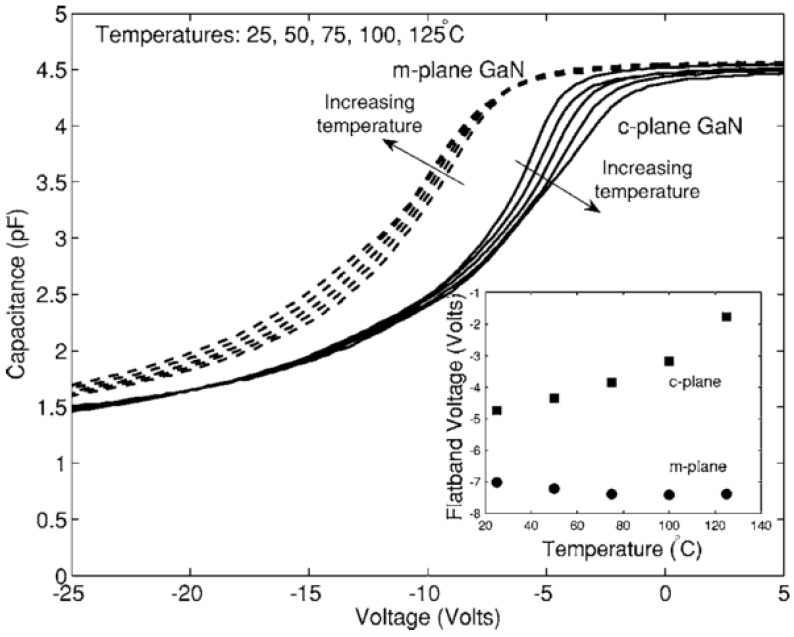
Examination of the pyroelectric effect on the capacitance voltage (CV) profile of GaN based devices [88]. Reprinted with permission from reference 88. Copyright (2007) by the American Institute of Physics.

1. Doping: In general for Si-SiO_2_ MOS capacitors, the doping concentration is modest, on the order of ~10^16^ cm^−3^. For GaN devices, there is significant variation in literature reports, with levels between ~10^16^ cm^−3^ and ~10^18^ cm^−3^ [85,86,87] This doping variation has a significant impact on the subsequent capacitance voltage profiles, with a more stretched-out profile observed in depletion going to deep depletion for a highly-doped channel layer. Qualitative analysis of such CV data might be misinterpreted as a high interface state density. An example of the effect of the GaN dopant concentration on measured capacitance voltage profiles is shown in [87].
2. Oxide thickness: Metal-oxide-semiconductor structures for conventional CMOS digital applications require thin dielectrics. The push for < 1 nm equivalent oxide thicknesses (EOT - the thickness of SiO_2_ that would be required to achieve the same capacitance as the gate stack with adielectric other than SiO_2_) has been the focus of much investigation for highly-scaled MOS devices. However, for GaN based devices used in high power and high temperature applications, the oxide thicknesses employed have thus far been much thicker—up to 150 nm—compared to those investigated in CMOS logic [88]. Recent research has begun to focus on thinner oxides [89]. When working with thick oxides, the interpretation of the capacitance voltage profile can be complicated by the fact that the electric field in the GaN is reduced and, for the same interface state density, the modulation of the surface charge on the semiconductor—and thus the Fermi level movement—by the AC signal of the CV meter will be reduced. This leads to a more stretched out capacitance-voltage profile, which could be misinterpreted as a higher D_it_.
3. Extraction of D_it_: In materials with spontaneous polarization charges, such as the Group 3-nitrides, the Gray–Brown technique [90] is not useful because the effect of the interface traps on the flatband voltage shift as temperature is varied is confounded by the presence of pyroelectric polarization charges (the change in spontaneous charge density with temperature) [91]. There are two difficulties in applying the conventional Terman method [92] for D_it_ measurements of GaN-based MOS devices. Firstly, it fails to describe interface states with energies in portions of the band gap that are not close to the GaN conduction band edge due to the extremely low hole generation rate (as a result of the large band gap of GaN). Once an interface state captures an electron in this system, the lack of holes does not allow the electron to recombine [85]. The Terman method relies on the states to emit and/or capture carriers at high frequency while the DC bias is swept. If recombination can happen, the traps respond to the slowly varying DC gate voltage and cause the CV profile to “stretch out” along the gate voltage axis as interface trap occupancy changes with gate bias. Secondly, in the estimation of D_it_ using the conventional Terman method, doping concentration should be known exactly to compare the difference between the measured and calculated CV profiles. However, doping concentration for a calculated CV profile is selected until a close fit is obtained over the entire voltage range, because the CV characteristics of GaN MOS capacitors show deep depletion instead of inversion at practical measurement temperatures. Therefore, the “stretch-out” of the CV associated with uniformly distributed D_it_ can be misinterpreted as an increased doping concentration, leading to an underestimation of D_it_ [93]. The high-low frequency method or conductance method, which do not require a theoretical profile to compare with the measured profile, can be considered to reduce the uncertainty associated with doping concentration in CV characterizations of the GaN MOS system. However, due to high series resistance and system noise, very few quasi-static or low (below 100 Hz) frequency CV profiles have been reported to date [94] and the conductance method can only measure interface states with short emission times [85]. The method developed for SiC—a photo-assisted CV measurement—was used by Wu *et al.* on GaN [95] and measurements taken at varying temperatures were demonstrated in references [96] and [97]. However, photo-assisted high-frequency CV only yields the average interface state density as derived from the total number of interface states divided by the bandgap energy range. Swenson *et al.* [85] have modified the photo-assisted procedure to determine the interface state density variation across most of the GaN bandgap, by comparing an ‘ideal’ CV in the dark with a CV measured following UV illumination, see Figure 8. This procedure was optimized for the Si_3_N_4_/GaN system. More work is required in order to develop a method or a complimentary range of methods including deep level transient spectroscopy (DLTS), deep level optical spectroscopy (DLOS) as well as modified versions of current conventional CV/GV methods described herein for use on GaN-based MOS devices. In the summary of GaN MOS device performance presented in Table 3, the method used to extract the interface state density (if known) is stated and the limitations of the various methods should be borne in mind when interpreting the data.
4. Pyroelectric effect: The pyroelectric effect is the change in spontaneous charge density with temperature that occurs in crystals that lack a center of symmetry. Pyroelectricity is one source of the strong temperature shift in flatband voltage and threshold voltage of GaN MOS capacitors and MOSFETs. Matocha *et al.* [88] have demonstrated a clear effect of the pyroelectric charge on MOS capacitor characteristics (see Figure 9). This complicates the interpretation of measurements with varying temperature [96,98] as any interface state density response will also vary with varying temperature.

### 4.2. Choice of Gate Insulator Material and Preferred Deposition Technique

**Figure 10:**
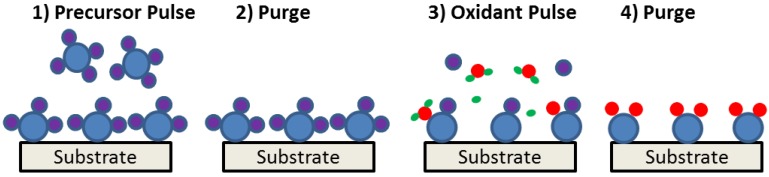
Schematic representation of atomic layer deposition process.

For any gate insulator candidate being considered, there should be a sufficient conduction band (for n-MOS devices) or valence band (for p-MOS devices) offset to the GaN to ensure a significant barrier height (> 1 eV) for low gate leakage currents. Also, in terms of future integration into manufacturing processes, it may be beneficial for the adopted dielectric to be deposited by atomic layer deposition (ALD), which is now widely implemented by the semiconductor industry for transistor gate dielectrics and damascene Cu metallization liners in silicon integrated circuits [99,100,101]. Atomic layer deposition is a self-limiting vapor-phase thin film deposition method. In ALD of metal oxides, one growth cycle consists of an exposure to metal precursors (or oxygen precursors), a purge period, an exposure to oxygen precursors (or metal precursors), and a second purge period, illustrated in Figure 10. During the first step, metal precursors are introduced into the reactor and adsorbed on the sample surface by reacting with surface functional groups. This, in general, results in less than monolayer coverage of the sample surface with each precursor pulse. Then, excess precursors are removed from the reactor by an inert gas purge. Following the second purge, an oxidant is introduced and reacts with the new surface functional groups formed from the previous pulse. Common oxidants include H_2_O or O_3_ in a thermal ALD process, or atomic oxygen in a plasma ALD process. Once the reaction is completed, the excess reactants are purged from the reactor. By this purging process, excess reactants do not contribute to additional film growth. This cyclic self-terminating process enables accurate control of the film thickness, large area film uniformity, highly conformal and pinhole-free film deposition.

### 4.3. MOS Devices Reported

Silicon dioxide and SiN_x_ are the most frequently reported dielectrics on GaN that are currently of interest [86,96,102]. The band offsets for both SiO_2_ and SiN_x_ on GaN have been determined by Cook *et al.* [103,104]. For SiO_2_ on GaN, the determined valence band offset is 2 eV and the conduction band offset is 3.6 eV. For SiN_x_, the determined conduction band offset is 2.4 eV but the valence band offset is −0.6eV. A remote plasma assisted oxide treatment of the GaN surface prior to SiO_2_ deposition is reported to result in a near ideal capacitance voltage profile [105] and a reduced interface state density [106]. The aim of this type of treatment is to create a high quality Ga-oxide interlayer to allow for a more favourable transition (fewer electrically active defects) to the deposited dielectric of choice. Variations of this approach include Ga_2_O_3_(Gd_2_O_3_) on GaN [107], GaO on GaN [108], as well as n-GaN/nitrided-thin-Ga_2_O_3_/SiO_2_ devices [109]. However, the small conduction band offset between Ga_2_O_3_ and GaN severely limits its value as a gate insulator. The authors of reference [91] showed that the SiO_2_-GaN interface can withstand annealing temperatures up to 1100^o^C in N_2_ – which is useful for gate-first transistor fabrication processes. For this SiO_2_-GaN device, the interface-state density increases approaching 1 eV below the conduction band edge, which is correlated with an increasing dispersion observed in the CV profiles with decreasing frequency and increasing temperatures. The authors of [91] note that increasing interface-state density deeper in the band gap was also observed in GaN MOS capacitors fabricated with a silicon oxide/nitride/oxide stack. This suggests that SiO_2_ is not an ideal candidate for inversion mode MOSFETs. Interestingly silicon nitride on GaN (0001) is reported to have the opposite D_it_ distribution, decreasing deeper into the bandgap [85,109].

**Figure 11:**
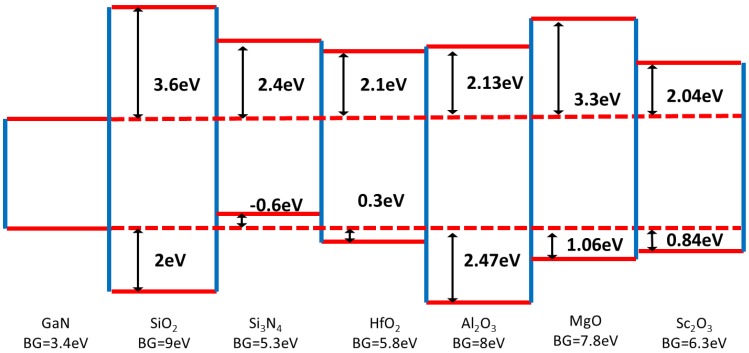
Conduction and valence band offsets for varying dielectrics on GaN determined experimentally from references [103,104,111,112,115,116].

Alternative dielectric materials have also been explored. A high dielectric constant (κ) layer as the gate oxide material to replace SiO_2_ (or SiN_x_) allows the physical thickness of the oxide to be increased, thereby reducing the gate leakage current, while maintaining or increasing capacitance per unit area of the gate oxide. Magnesium oxide and scandium oxide were investigated in reference [110]. Their dielectric constants (~ 9 for MgO and 14 Sc_2_O_3_) and their ability to function as a regrowth mask for selective area regrowth of GaN are advantageous. The conduction band and valence band offsets of MgO on GaN are 3.3eV and 1.06eV respectively, determined experimentally in [111]. The conduction band and valence band offsets of Sc_2_O_3_ on GaN were determined experimentally to be 2.04 eV and 0.84 eV [112]. Aluminium oxide and hafnium oxide dielectrics on GaN have recently been the subject of several literature reports. These metal oxides are readily deposited by atomic layer deposition (ALD). One of the first reported studies of atomic layer deposited Al_2_O_3_ on GaN MOS devices was by Wu *et al.* [95]. Using the photo-assisted CV method, the minimum average interface state density reported by these authors was 7 × 10^10^ cm^−2^ eV^−1^, following a post-Al_2_O_3_ deposition 800^o^C RTA in N_2_. An early report focusing on HfO_2_ MOS devices on GaN was published in the same year by Chang *et al.* [113]. They showed that the ALD process for HfO_2_ (using tetraethylmethylamido hafnium as the metal precursor) resulted in an interfacial layer between the oxide and the semiconductor which they identified as gallium oxynitride by x-ray photoelectron spectroscopy (XPS). This is in contrast to Al_2_O_3_ deposition by ALD on other 3-V surfaces such as GaAs and InGaAs, where the initial stages of the process is often reported to result in the decomposition of native oxides formed on the semiconductor surface, with trimethylaluminium (TMA) as the metal source [114]. This phenomenon is routinely referred to as a ‘self-cleaning’ process. Using the Terman method, a D_it_ of 2 × 10^11^ cm^−2^ eV^−1^ at midgap was reported in [113]. Cook *et al.* [115] determined the band offsets for HfO_2_ on GaN, with the HfO_2_ formed by remote plasma oxidation of monolayers of deposited Hf. The determined conduction band offset was 2.1 eV and the valence band offset was 0.3 eV. More recently, the conduction band offset of Al_2_O_3_ on GaN was determined experimentally to be 2.13 eV, in agreement with theoretical predictions [116]. Figure 11 illustrates the conduction and valence band offsets of the afore mentioned dielectrics to GaN.

**Figure 12:**
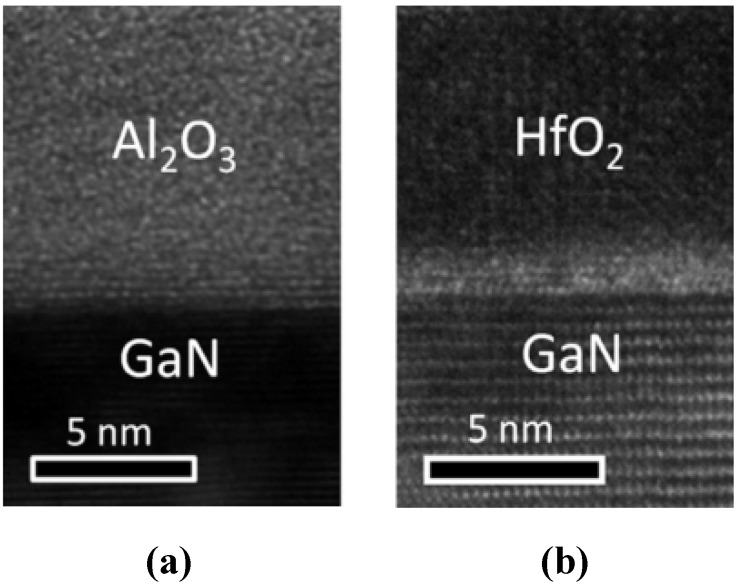
Al_2_O_3_ and HfO_2_ deposited by ALD on GaN for (**a**) Al_2_O_3_ showing no interlayer; and (**b**) HfO_2_ on GaN showing an interlayer [89]. Reprinted with permission from reference [89]. Copyright (2011) by the Elsevier.

Chang *et al.* reported on ALD Al_2_O_3_ on p-type GaN with a near-midgap D_it_ of 4–9 × 10^11^ cm^−2^ eV^−1^, using the conductance method. In the same year, reference [117] reported a higher interface state density at the conduction band edge for the ALD Al_2_O_3_-GaN interface, with decreasing density further into the band gap. Using the photo-assisted CV method, those authors found the interface state density to be ~ 1 × 10^12^ cm^−2^ eV^−1^ near the conduction band edge. Interestingly this is similar to the D_it_ profile for Si_3_N_4_ on GaN discussed earlier. In 2010, Chang *et al.* reported an interface state density to be around 5–8 × 10^11^ cm^−2^ eV^−1^ near the conduction-band minimum of GaN using the conductance method [22]. Chang *et al.* [89] reported a systematic study of Al_2_O_3_/GaN, HfO_2_/GaN and HfO_2_/Al_2_O_3_/GaN capacitors. In contrast to their previous work, where they detected an interfacial layer between the ALD HfO_2_ and GaN, in this paper they report an abrupt interface between the Al_2_O_3_ and the GaN by transmission electron microscopy (TEM) observation. Figure 12 compares the resulting interfaces. Using the conventional Terman method, they extracted the interface state density near midgap, in which there were minor variations between samples in the range 5–11 × 10^11^ cm^−2^ eV^−1^. In comparison, reference [73] suggests that the ‘self-cleaning’ effect of the aluminium ALD precursor, TMA, as observed on InGaAs and GaAs, does not occur on GaN. In an alternative approach, the authors of [70] showed that deposition of an Al metal layer in vacuum using a Knudsen cell onto the air-exposed GaN surface caused interfacial reactions, resulting in the formation of oxide layers including Al_2_O_3_ and Ga oxide at the interface. On p-type GaN, Lee *et al.* have investigated two nanolaminate stacks composed of Al_2_O_3_/TiO_2_/Al_2_O_3_ and MgO/TiO_2_/MgO [118,119] enabling a higher capacitance density while maintaining low gate leakage. They report that the best performance from both of these stacks is achieved when the GaN surface is treated with aqueous (NH_4_)_2_S_x_, and a post metal anneal in N_2_. They report a D_it_ at midgap of ~ 4 × 10^11^ eV^−1^ cm^−2^ for the Al_2_O_3_/TiO_2_/Al_2_O_3_ stack and ~ 3 × 10^11^ eV^−1^cm^−2^ for the MgO/TiO_2_/MgO gate stack, using the Terman method.

There are a number of points of interest summarized in Table 3:

1. It is clear that the possible dielectrics being adopted for GaN go beyond the SiO_2_ and SiN_x_, with atomic layer deposition being the technique of most interest recently (and possibly most relevant to manufacturing).
2. It is also clear that the surface cleaning treatments being used vary widely – from *in situ* vacuum anneals, to *ex situ* acid and/or base treatments.
3. The method for extraction of the interface state density and the densities themselves also vary widely—in general the values presented when extracted using the Terman method are lower. With such variance in the determination of D_it_, and the difficulty of quantifying D_it_ in a relatively large band gap system, it is very difficult to draw solid conclusions from the values presented, and thus to concretely deduce more favorable treatments and dielectric deposition techniques.

**Table 3:** Summary of relatively recent literature on the electrical characteristics of GaN MOS devices.

Reference	GaN growth technique	Dielectric	Deposition technique	GaN clean/treatment	*D*_it_ extraction method	*D*_it_ cm^−2^ eV^−1^
[89]	MOCVD	Al_2_O_3_, HfO_2_	ALD	None mentioned	Terman	Near Midgap 6–10 × 10^11^
[94]	MOCVD, MBE	SiO_2_	(Electron Cyclotron Resonance-Plasma Enhance CVD)	UV/Ozone oxidation, BOE; thermal anneal at 300°C in UHV	High-low method	2 × 10^11^
[120]	–	Al_2_O_3_, HfO_2_	ALD	None mentioned	Conductance Technique	~ 10^12^
[97]	–	HfAlO	MOCVD	10 min HCl (H_2_O/HCl = 1:1)(NH_4_)_2_S solution for 30 min	Conductance with increased temperature	At midgap: As deposited 2 × 10^12^; with SiH_4_+NH_3_ 2 × 10^10^
[121]	MOCVD	Al_2_O_3_	ALD	30% HF	Terman	< 1 × 10^12^
[122]	MOCVDP Type	Oxidized GaN	bias-assisted photo electrochemical oxidation	BOE	Photo assisted CV	Average value 4.18 × 10^11^
[86]	CVDP Type	SiO_2_	Plasma Enhance CVD	RCA-based cleaning process	Terman	1.1 × 10^11^ and 6 × 10^11^ at E_c_ − E_T_ = 0.2 eV for silane & TEOS; 1.1 ×10^11^ and 1 × 10^12^ at E_T_−E_V_ = 0.2 eV for silane and TEOS
[85]	MOCVD	Si_3_N_4_	MOCVD	None mentioned	Modified Photo assisted CV	5.0 × 10^12^ at 0.3 eV
[117]	MOCVD	Al_2_O_3_	ALD	H_2_SO_4_:H_2_O_2_ (3:1) for 5 min and HCl:H_2_O (1:1) for 3 min	Photo assisted + Conductance Technique	3 × 10^12^ as deposited
[123]	MOCVDP Type	Al_2_O_3_, HfO_2_	ALD	HCl	Conductance Technique	4–9 × 10^11^ near midgap
[98]	MOCVD	Al_2_O_3_	ALD	None	Terman	1 × 10^13^ at CB edge
[107]	MBE	Ga_2_O_3_(Gd_2_O_3_)	Ebeam	600 °C UHV	Terman	1 × 10^11^ at midgap,
[113]	MOCVD	HfO_2_	ALD	None	Terman	2 × 10^11^ at midgap
[95]	MOCVD	Al_2_O_3_	ALD	RCA-based cleaning process	Photo assisted Technique + Terman	Midgap: Terman 5 × 10^10^ Photoassisted ~ 5 × 10^11^
[124]	MOCVDN and P Type	SiO_2_	Plasma Enhanced CVD	(1:1 H_2_SO_4_/H_2_O_2_) for 10 min then (1:3 HCl/H_2_O) for 3 min	Conductance Technique	6 × 10^10^ at E_c_ − 0.2 eV; 1.4 × 10^11^ (at 0.61 eV) near the valence band; 7.5 × 10^10^ deeper into the band gap.
[106]	HVPE	SiO_2_	Remote Plasma Assisted Oxidation/Remote Plasma Enhanced CVD	None mentioned	Terman	Min D_it_ 1 × 10^11^ at ~ 0.45 eV below CB
[93]	HVPE	SiO_2_	Remote Plasma Assisted Oxidation (Nitridation)	1:5 NH_4_OH:H_2_O at 60–80 °C (or in HCl based solutions).	Not Stated	Midgap 10^11^
[109]	MOCVD	SiO_2_; SiN_x_	Remote Plasma Enhance CVD	NH_4_OH/H_2_O 1:5 solution at 80 °C for 15 min	High-low and Conductance	mid 10^11^ at E_C_-E ~ 0.3 eV
[91]	MOCVD	SiO_2_	Low Pressure CVD	H_2_SO_4_ : H_2_O_2_ (1:1) at 70^o^C for 10 min	Conductance Technique	Best: 1 × 10^11^ at 0.25 eV below CB edge
[125]	HVPE	SiO_2_	Remote Plasma Assisted Oxidation	1:5 NH_4_OH:H_2_O solutions at 60–80 °C (or in HCl-based solutions). Exposed to reactive species from a remote N_2_/He discharge	Terman	Min D_it_: 3 × 10^11^ with RPAO 2 × 10^12^ without RPAO samples
[126]	MBE, MOCVD	SiO_2_	Plasma Enhanced CVD	1 HCl:2 H_2_O	Terman	Minimum for as deposited 2 × 10^11^
[118]	MOCVDP Type	Al_2_O_3_/TiO_2_/Al_2_O_3_	RF Sputtering	(NH4)2Sx	Terman	4 × 10^11^ Midgap
[119]	MOCVDP Type	MgO/TiO_2_/MgO	RF Sputtering	(NH4)2Sx	Terman	3 × 10^11^ Midgap

## 5. Discussion

### 5.1. Matching Reported GaN Bulk and Surface Defects to Reported MOS Characteristics

While attempting to link the identified defects and the corresponding Fermi level pinning position in Table I to the characteristics of electrically active defects inferred from data in Table 3, the following should be considered:

1. From Table 1, there are defect states related to C impurities in the GaN bulk that have energies near the valence band maximum, as do Ga vacancies in GaN.
2. From Table 1, Ga dangling bonds and Ga-Ga bonds have defect energies in the upper half and mid to lower half of the bandgap respectively.
3. From Table 1, there is a defect level close to the conduction band minimum resulting from N vacancies.
4. Combining the information from points 1, 2 and 3, suggests that mid gap, where many of the experimental D_it_ values in the literature are quoted, is actually where the interface trap density may be relatively low, giving a misleading view of the actual defect density.
5. The MOS device literature reviewed herein suggests that defects at the GaN surface do not typically pin the Fermi level, as the maximum capacitance reached experimentally is consistent with ideal predicted values. However, the fact that so many defects with energies near the valence band maximum are experimentally detected suggests a possible difficulty in making functioning inversion mode MOS devices on GaN.
6. The D_it_ values reported in the literature may reflect not only the influence of dielectric/GaN interface defects, but also of defects in the dielectric and in the near-surface region of the GaN substrate.

### 5.2. Defects at the Dielectric/GaN Interface

As mentioned in point 6 above, once a dielectric material has been deposited on the GaN surface, it is highly likely that defects will form at the dielectric/GaN interface. These defects are part of the overall defect density profile that is characteristic of the interface. The relationship between the interface state density distribution in the GaN band gap and the dielectric material could give further insight into the microscopic origin of the interface states. If the D_it_ profiles are largely similar for devices with varying gate dielectrics, it would suggest that the defects are intrinsic to the semiconductor surface (e.g., dangling bonds). However, if the interface defect profiles significantly differ as a function of the dielectric material, the dielectric deposition conditions and interfacial reactions may be the defining factor. Ostermaier *et al.* [117] reports the interface state density for their ALD-grown Al_2_O_3_ on n-GaN (0001) to be higher around the conduction band edge and decreasing deeper into the band gap. This result is similar to most reports for SiN_x_ dielectrics on GaN, in which the D_it_ is reported to increase as the Fermi level moves from the conduction band minimum towards midgap, as discussed above.

The mechanism by which the gate insulator layer on GaN substrate nucleates during the initial cycles of atomic layer deposition should also be considered. For example in reference [127], the authors report scattered 3D nucleation of ALD Al_2_O_3_ on HCl treated GaN, while piranha etch solution (H_2_O_2_:H_2_SO_4_ = 1:5) and HF pretreated GaN surfaces resulted in smooth Al_2_O_3_ layers. Also, as mentioned in Section 3, it is as yet not clear whether the ‘self-cleaning’ effect typically observed for ALD Al_2_O_3_ deposition on GaAs and InGaAs substrates also occurs for GaN.

### 5.3. Reduction of Defects at the Dielectric/GaN Interface

The interface state densities presented in Section 3 are quite high (using the relationship between D_it_ and the subthreshold slope of the transistor for a 20 nm film of Al_2_O_3_, a D_it_ lower than 1 × 10^12^ cm^−2^ eV^−1^ would be desirable to achieve a subthreshold slope of 100mV/decade or less [128]). Reduction of these defect densities is required for incorporation of the devices into commercial applications. Reduction of defects at the GaN-dielectric interface could be achieved by treating the GaN surface prior to dielectric deposition. At this point the reader might ask why the effects of the *ex situ* surface treatments discussed in Section 2 are not discussed in Section 3. This question points to a gap in the knowledge of the mechanistic effects of surface preparation on the subsequent electrical characteristics of GaN MOS devices. Most of the treatments in Section 2 are intended to form metal-semiconductor Schottky contacts rather than MOS devices. However, with a good base of data available, this previous work has value for MOS interface preparation. It is also important to note that across the literature referenced in Section 3, most of the MOS devices experience an anneal either during or after the surface treatments. Further research is needed on the specific effects of these anneals on the dielectric’s properties and its interface with GaN. In addition, the majority (but not all) of the work described in the literature is on n-type GaN channels. An optimized process for reduced interface state density at the oxide/n-type semiconductor interface is unlikely to be directly transferrable to a p-type GaN device. In fact, reference [129] demonstrates that for similar interface preparation steps for each GaN doping type (n-type, p-type and unintentionally doped), the optimization of the surface preparation is strongly dependent on the GaN doping type.

### 5.4. Defects in the Dielectric

**Figure 13:**
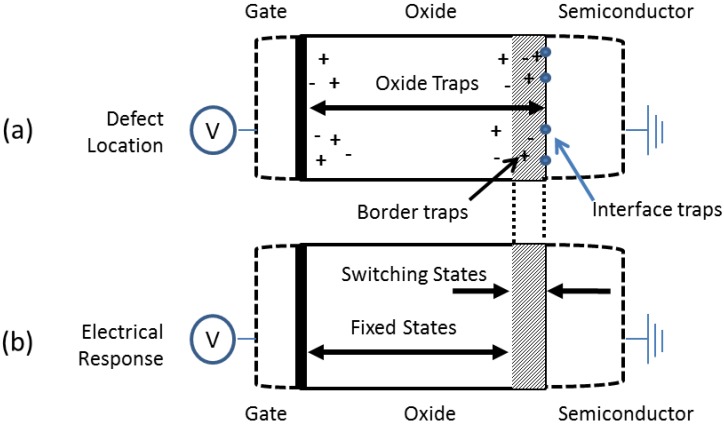
Schematic illustration of border traps.

In addition to defects present at the GaN-dielectric interface leading to defect energy levels in the GaN band gap, trap levels in the deposited dielectric which align in energy with the bandgap of the GaN must also be considered. ‘Border traps’ [130,131] are defects in the dielectric layer (but not at the semiconductor/dielectric interface), which can communicate with carriers in the underlying semiconductor (see Figure 13). They can be accessed via carrier tunneling through the interface into the dielectric from the semiconductor [132] and, therefore, their generation depends strongly on the quality and compositional abruptness of the semiconductor/dielectric interface. The probability of tunneling from the semiconductor into a trap falls off exponentially with increasing distance of the trap into the dielectric. Therefore, the depth into the dielectric at which traps will electrically respond is determined by the measurement conditions. Fewer oxide traps will exchange charge with the semiconductor during high frequency measurements than for low frequency measurements [131]. Their response will be frequency dependent but not temperature dependent [132], which helps to distinguish a border trap response from an interface state response. To date, border traps in GaN MOS devices have not been discussed in the literature.

A systematic study of the effects of substrate surface preparation and metal oxide dielectric growth on C-V hysteresis (shift of the C-V curve along the voltage axis due to slow charge trapping in the dielectric) in a given device is also needed. For high power (high voltage) devices, a large hysteresis of the threshold voltage under bias stressing can severely degrade performance. To date, no substantial study of hysteresis in GaN MOS devices has been reported, although Nepal *et al.* did do some direct comparisons of surface cleans in terms of surface roughness and the subsequent measured hysteresis [127]. The thermal stability of the dielectric materials themselves may cause their properties to drift during operation at higher temperatures for sustained periods of time [133]. Removal or formation of fixed interfacial and bulk charges and charge traps during high temperature stressing are well known in the high-κ gate oxide literatures for silicon devices [134,135,136]. Once the selection rules for deposited gate insulators on GaN-based devices are better understood, a new focus on reliability of MOS gate stacks on GaN power devices will be needed.

### 5.5. The MOS-HEMT

As previously mentioned, the AlGaN/GaN Schottky gate HEMT structure is also relevant for high frequency and high power applications. However, with the Schottky gate devices, a low Schottky barrier height and/or poor interface quality can lead to high gate leakage as well as low breakdown voltages. In order to address these two shortcomings in particular, some work has been reported on the deposition of high-κ materials on AlGaN/GaN structures. Such structures are referred to as MOS-HEMT structures. La_2_O_3_ [137], Pr_2_O_3_ [138], Ga_2_O_3_ [139], ZrO_2_ [140] and HfO_2_ [15,139,141] have all been reported, however it is Al_2_O_3_ which is the most widely investigated candidate to date [14,18,139,141,142,143,144]. For all of these dielectrics, reduced gate leakage of the MOS-HEMT compared to that of the Schottky HEMT is reported, with additional benefits such as higher drain current, increased immunity to current collapse, increased transconductance and higher breakdown voltage also reported [14,15,18,137,138,139,140,141,142,143,144,145,146]. Once again, ALD shows promising results with one recent report demonstrating reduced trap states at the Al_2_O_3_/AlGaN interface when compared to MOCVD-grown Al_2_O_3_ or oxidation of a thin Al surface layer to form Al_2_O_3_ [14]. As mentioned in the introduction, some HEMT structures have GaN capping layers on the AlGaN surface [14,142,143] and thus the learning obtained from GaN MOS devices can also be used for the improvement of MOS-HEMT devices. In fact, some reports suggest that the Al_2_O_3_/GaN interface has reduced trap states as compared to the Al_2_O_3_/AlGaN interface [14,142].

## 6. Conclusions

The literature on GaN surfaces, surface treatments and gate dielectrics relevant to metal oxide semiconductor devices has been reviewed. In Section I, the significance of the GaN growth technique and growth parameters on the properties of GaN epilayers was summarized. In Section 2, the ability to modify GaN surface properties using *in situ* and *ex situ* processes was demonstrated. In Section 3, progress on the understanding and performance of GaN MOS devices was presented and discussed. Although a reasonably consistent picture is emerging from focused studies on topics covered in each of these three Sections, future research can achieve a better understanding of the critical oxide-semiconductor interface by probing the connections between these topics. In addition, this review highlighted the need to develop more rigorous and quantitative methods for analyzing defect concentrations and energies in GaN MOS gate stacks. Promising gate dielectric deposition techniques such as atomic layer deposition, which is already accepted by the semiconductor industry for silicon CMOS device fabrication, coupled with more advanced physical and electrical characterization methods will likely accelerate the pace of learning required to develop future GaN-based MOS technology.
